# The effect of rituximab on patient reported outcomes in the preclinical phase of rheumatoid arthritis: 2 year data from the PRAIRI study

**DOI:** 10.1136/rmdopen-2024-004622

**Published:** 2024-10-18

**Authors:** Giulia Frazzei, Sophie H M Cramer, Robert B M Landewé, Karen I Maijer, Danielle M Gerlag, Paul P Tak, Niek de Vries, Lisa G M van Baarsen, Ronald F van Vollenhoven, Sander W Tas

**Affiliations:** 1 Department of Rheumatology and Clinical Immunology, Amsterdam University Medical Center, University of Amsterdam, Amsterdam, The Netherlands; 2 Amsterdam Rheumatology and Immunology Center, Amsterdam, The Netherlands; 3 Rheumatology, Zuyderland MC, Heerlen, The Netherlands; 4 Dermatology, Tergooi Hospital, Hilversum, The Netherlands; 5 UCB Pharma Ltd, Slough, UK; 6 Candel Therapeutics, Needham, Massachusetts, USA; 7 Research and Development, GSK, Stevenage, UK

**Keywords:** Arthritis, Rheumatoid, Rituximab, Patient Reported Outcome Measures

## Abstract

**ABSTRACT:**

**Objectives:**

Early treatment of individuals at risk of developing rheumatoid arthritis (RA-risk) in the preclinical phase has the potential to positively impact both patients and society by preventing disease onset and improving patients’ quality of life. The PRAIRI study was a randomised, double-blind, placebo-controlled trial with the B-cell depleting agent rituximab (RTX), which resulted in a significant delay of arthritis development of up to 12 months in seropositive RA-risk individuals. Here, we report our findings on patient-reported outcomes (PROs) in this study population.

**Methods:**

Seventy-eight RA-risk individuals were treated with one single dose of either placebo (PBO) or 1000 mg RTX plus 100 mg methylprednisolone (MP) and anti-histamines, regardless of treatment allocation, as co-medication. Data on quality of life were collected at baseline and 1, 4, 6, 12 and 24 months using established PRO questionnaires (visual analogue scale (VAS) pain, health assessment questionnaire disability index (HAQ-DI) score, EuroQol five dimension (EQ-5D) and both physical and mental component score of the 36-item short-form heath survey (SF-36)).

**Results:**

No significant changes in quality of life over a 2 year follow-up were observed in at-risk individuals treated with RTX compared to PBO given the PRO scores at 24 months (mean difference±SEM: HAQ score=0.07±0.16; EQ-5D=−0.02±0.05; VAS pain=11.11±7.40). Furthermore, no significant effect of treatment on perceived arthritis severity at the time of clinically manifest disease (arthritis) was found.

**Conclusion:**

One single dose of RTX plus MP administered to RA-risk individuals does not have a meaningful and measurable positive effect on PROs after 2 years of follow-up and/or perceived disease severity at the time of arthritis development.

**Trial registration number:**

Trial registered at EU Clinical Trial Register, EudraCT Number: 2009-010955-29 (https://www.clinicaltrialsregister.eu/ctr-search/search?query=Prevention+of+RA+by+B+cell+directed+therapy).

**WHAT IS ALREADY KNOWN ON THIS TOPIC**

- B cells are involved in rheumatoid arthritis (RA) pathogenesis, and their depletion in early and established RA leads to improved clinical outcomes, radiographic progression, and physical functioning.
- In the PRAIRI study, B-cell depletion by a single dose of 1000 mg rituximab (RTX) in RA-risk individuals led to 55% decreased risk of arthritis development at 12 months compared with placebo (PBO), with a 12 month delay of disease onset.
- Preventive treatment with methotrexate and abatacept in RA-risk individuals lead to decreased disease burden, with sustained effect after treatment, even when prevention was not achieved.

**WHAT THIS STUDY ADDS**

- We investigated the effects of a single dose of 1000 mg RTX on patient perception of disease burden by measuring various patient-reported outcomes (PROs).
- A single injection of RTX did not improve PROs on quality of life, pain, and physical functioning nor did it modulate perceived disease activity at the onset of arthritis.

**HOW THIS STUDY MIGHT AFFECT RESEARCH, PRACTICE OR POLICY**

- A single injection of RTX is not recommended to prevent arthritis or improve quality of life in RA-risk individuals.

## Introduction

 Over the past 10 years, the identification of individuals at risk of developing rheumatoid arthritis (RA-risk) in the preclinical phase—i.e. before the disease has been fully established—via the presence of autoantibodies (i.e. anti-citrullinated protein antibodies (ACPA) and/or immunoglobulin M (IgM)-rheumatoid factor (RF)) in combination with preclinical manifestations (i.e. arthralgia, morning stiffness, subclinical inflammation of the joints) has led to the initiation of preventive strategies with the potential to prevent disease or delay its onset, which may possibly result in a positive impact on both patient and society.

Considering the presence of high levels of IgA^+^ plasmablasts in the peripheral blood of RA-risk individuals and the development of anti-modified protein antibodies (AMPA) against acetylated and carbamylated peptides closer to disease onset, as well as changes in the BCR repertoire years before the disease onset and the localization of both B cells and plasma cells in the synovial tissue of early RA patients, it has been postulated an alteration of B-cell lineage cells in RA-risk individuals.^1^,^4^ The anti-CD20 monoclonal antibody rituximab (RTX) is an approved and effective drug for the treatment of RA, which has shown to decrease disease signs and symptoms in patients refractory to conventional and other biological disease-modifying anti-rheumatic drugs (DMARDs).^5^,^7^ Repeated treatment with RTX plus methotrexate (MTX) in early RA patients leads to improved clinical outcomes and reported physical functioning at the 2 year follow-up.^8^,^10^

The PRAIRI study was a randomised double-blind, placebo-controlled, phase II clinical trial, which postulated a possible role of B cells as potential therapeutic or preventive target in RA-risk individuals and investigated for the first time the effect of B-cell depletion in this population.^11^ A single infusion of 1000 mg RTX versus placebo (PBO) was administered to 81 seropositive individuals with arthralgia. RTX treatment resulted in B-cell depletion and a median delay in arthritis onset of up to 12 months at the point when 25% of subjects in both groups developed RA. Although the overall risk of developing RA at the end of follow-up was not significantly different, RTX decreased the risk of developing RA at 12 months by 55%.^11^

Besides the chance of achieving true prevention, another important argument in favour of interventions in RA-risk individuals may be the potential reduction in disease burden for individuals for whom preventive treatment is not effective. Patient-reported outcomes (PROs) are valuable tools to interrogate the impact of disease burden on patients. Recently, several RA prevention trials (i.e., TREAT-EARLIER, ARIAA, and APIPPRA) reported a significant improvement in PROs after treatment with MTX or abatacept, respectively, regardless of the efficacy of treatment in RA prevention.^12^,^14^ Both the TREAT-EARLIER and ARIAA trial reported sustained improvement of PROs after treatment cessation.^13 14^ Here, we investigated the effect of a single 1000 mg dose of RTX on quality of life in RA-risk individuals throughout 2 year follow-up time of the PRAIRI study. We hypothesised that RTX treatment would have a positive effect on perceived physical functioning and health-related quality of life.

## Methods

Between January 2010 and December 2013, 81 subjects with arthralgia who were positive for both IgM-RF and ACPA were included in the PRAIRI study and treated with 100 mg methylprednisolone and either one single dose of 1000 mg RTX or PBO. The clinical and cellular results from the PRAIRI study were published in 2019.^11^ As part of the study protocol, data on subjects’ quality of life, pain and physical functioning were collected at baseline and 1, 4, 6, 12, 24, 36, and 48 months with the use of the following questionnaires: health assessment questionnaire disability index (HAQ-DI) score, EuroQol five dimension (EQ-5D) questionnaire, visual analogue scale (VAS pain) and both the physical component score (PCS) and mental component score (MCS) of the 36-item short-form heath survey (SF-36).

Paper questionnaires were entered manually into electronic forms in Castor database, and data entry was independently checked randomly by two study researchers (GF and SHMC). In case of unclear or ambiguous answers in the paper files, data were reported as missing. After data entry completion, all data were extracted in IMB SPSS Statistics 28 and statistical analysis were performed in GraphPad prism 9. Participants were excluded by the analysis if baseline data were missing. Repeated measurements were analysed with a mixed-effect model to account for missing values; PROs at baseline versus arthritis were compared by independent T-test. Separate analyses were also performed when comparing outcomes at baseline versus 24 months. The mixed effect model was a random intercept and slope model without additional interaction terms but with time as a variable. Since there were no time-trends, we refrained from doing time-lagged analyses. The final model was the model that had lowest AIC. Statistical analyses were considered significant when p-values were <0.05.

HAQ-DI score was automatically calculated at the end of the questionnaire within the Castor dataset, according to the HAQ-DI guidelines.^15^ Quality of life data were collected through the EQ-5D, three-level questionnaire. Data were converted into an index (scale 0–1) using the validated reverse crosswalk analysis syntax available on the EuroQol website, with values adapted to the Dutch population.^16^ The aggregated data from SF-36 sub-categories (physical functioning, vitality, body pain, role limitations caused by physical problems, role limitations caused by emotional problems, general mental health, social functioning and perception of general health) were transformed into Z-scores using Dutch population mean and SD scores as reference; PCS (scale 1–100) and MSC (scale 1–100) were then calculated according to the instruction from the SF-36 user manual from Z-score values.^17 18^

### Patient and public involvement

Patients research partners were involved in the original design of the PRAIRI trial (a.o. via focus groups), as well as in dissemination of the previously reported clinical results. We will also involve our patient research partners in the dissemination of the current PRO data and any follow-up research.

## Results

### Study population and follow-up

**Figure 1:**
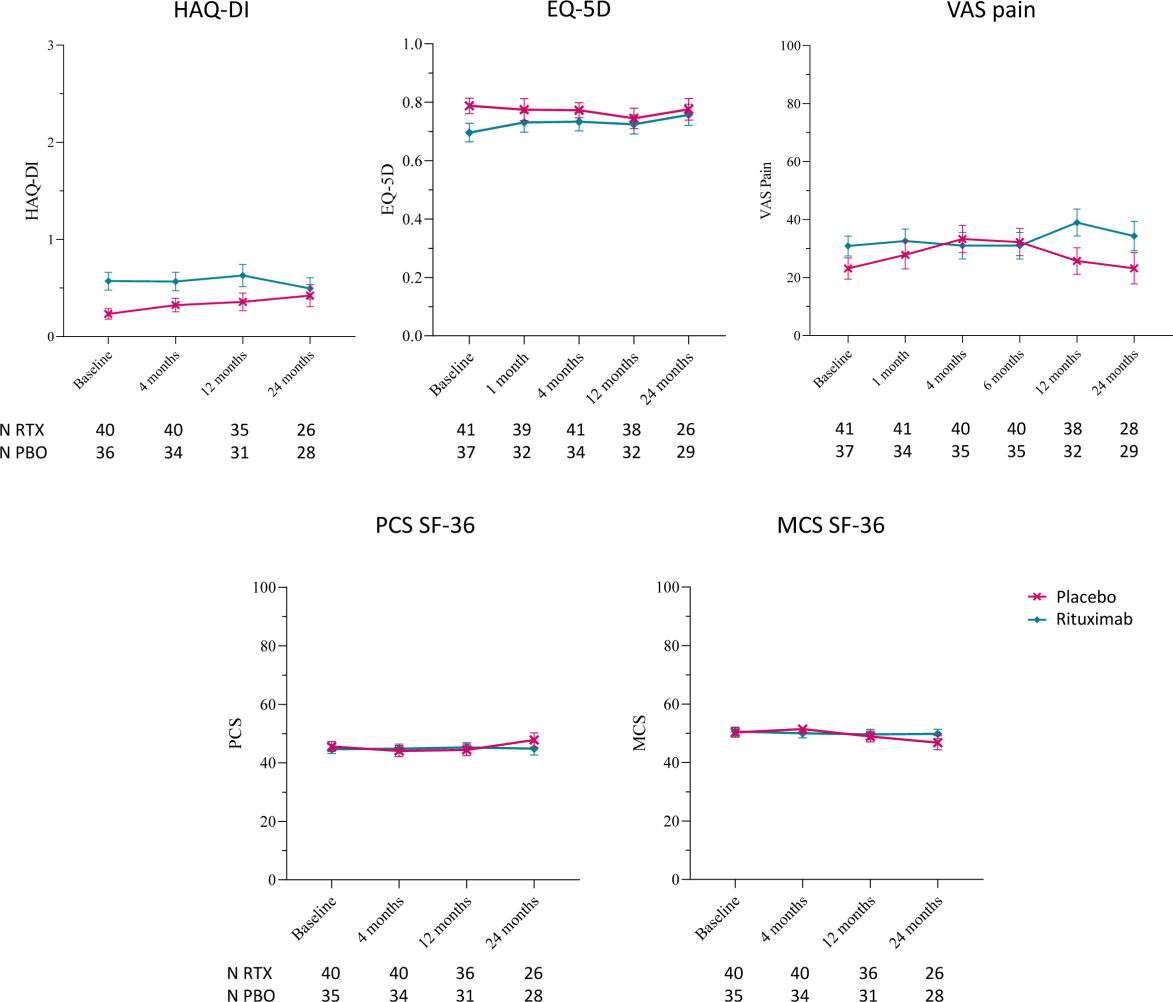
Patient reported outcomes (PROs) for the 2 year follow-up after a single infusion of rituximab or placebo. PRO scores for health assessment questionnaire disability index (HAQ-DI) score, EuroQol five dimension (EQ-5D) questionnaire, visual analogue scale (VAS) pain, physical component score (PCS) and mental component score (MCS) of 36-item short-form heath survey (SF-36) questionnaire, stratified for treatment (n at baseline: placebo (PBO)=37; rituximab (RTX)=41), reported as mean±SEM. PRO scores are shown for a 2 year period (PBO=red line; RTX=blue line).

Out of 81 patients, PROs from 78 patients, 41 in the RTX group and 37 in the PBO group, were available. Of these participants, 28 (68%) and 23 (62%) were women, respectively. Median age within groups were 53 (45-58) in the RTX group and 51 (42-58) in the PBO group. Median follow-up time was 24 (24–36) months in the RTX group and 25 (24–36) months in the PBO group; due to the high number of missing values at 36 and 48 months, analysis focused on the PROs up to 2 years after treatment. Two participants were excluded from further analysis in those categories on PCS and MCS of SF-36 due to missing data at baseline. Numbers of participants at each timepoint, stratified based on questionnaire and treatment, are reported in figure 1.

Fourteen subjects (34.14%) developed RA in the RTX group and 15 individuals (40.54%) in the PBO group. Median time to onset of arthritis was 17 (10–28) months in the RTX group and 12 (2–15) months in the PBO group. Data on baseline and at least one follow-up visit were needed for subjects to be included in the analysis. When comparing baseline values to the visit at the onset of arthritis, two individuals of the RTX-treated group were excluded from analysis due to missing PRO data at the onset of arthritis visit. One additional patient had missing HAQ-DI score at the onset of arthritis visit and one patient had missing baseline SF-36; these patients were excluded from analysis for HAQ score and SF-36, respectively. One patient of the PBO group was excluded from the analysis due to missing PROs at the onset of arthritis visit.

### Longitudinal changes in PROs during study follow-up and at RA onset

PRO scores did not significantly change during the 2 year follow-up period, both in the PBO and RTX groups, independently from arthritis development (figure 1). We observed a small difference in the HAQ-DI score between PBO (mean±SEM=0.23 ± 0.05) and RTX (mean±SEM=0.57 ± 0.09) at baseline, as well as slightly worse EQ-5D (mean±SEM: PBO=0.79 ± 0.03; RTX=0.70 ± 0.03) and VAS pain (mean±SEM: PBO=23.11 ± 3.68; RTX=30.88 ± 3.41) scores in the RTX group at baseline. Online supplemental table 1 reports the mean difference and 95% CI for each variable at each timepoint, based on the mixed effect model outcomes.

**Figure 2:**
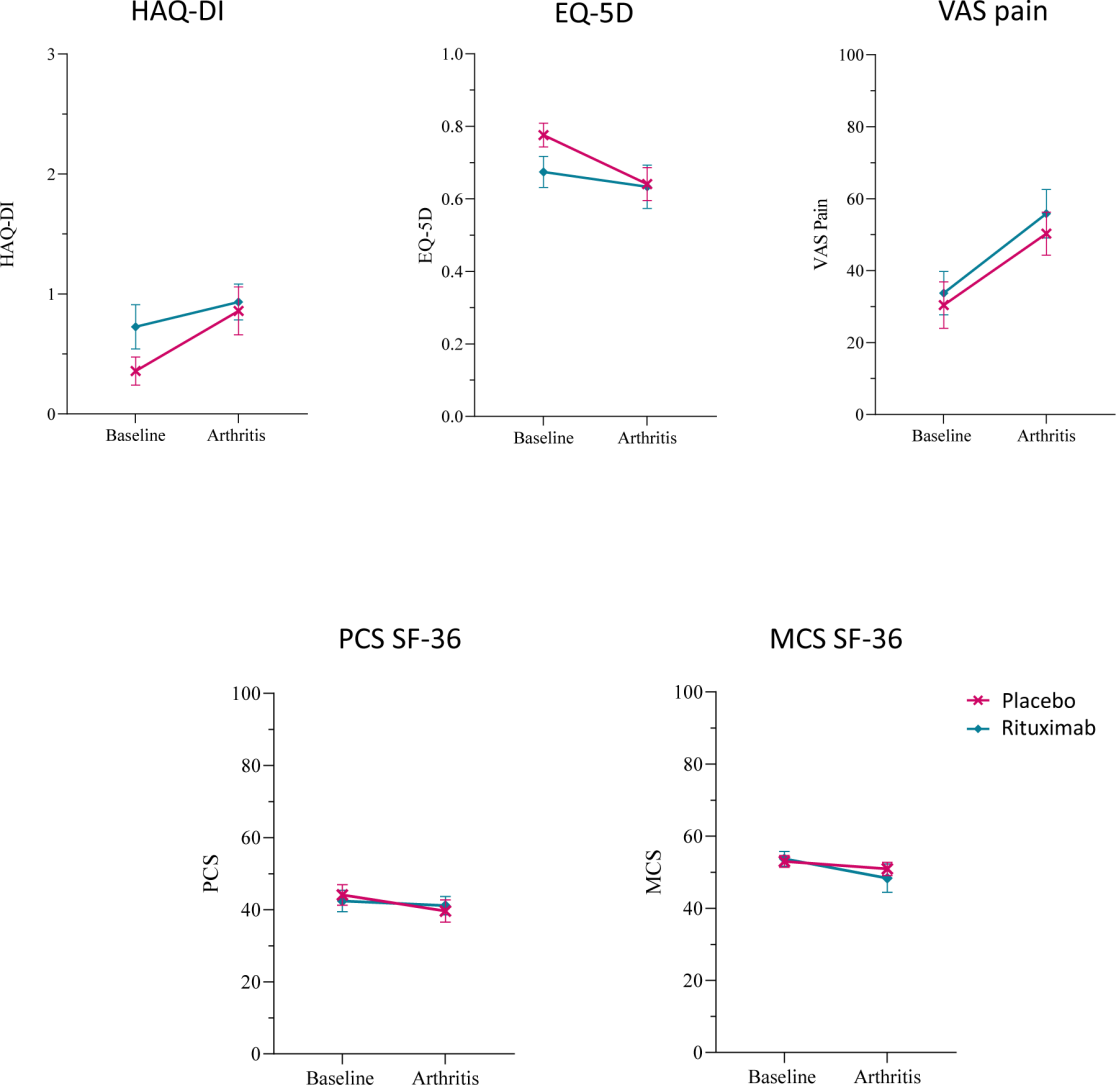
Patient-reported outcomes (PROs) in patients who developed arthritis. PRO scores for health assessment questionnaire disability index (HAQ-DI) score, EuroQol five dimension (EQ-5D) questionnaire, visual analogue scale (VAS) pain, physical component score (PCS) and mental component score (MCS) of the 36-item short-form heath survey (SF-36) questionnaire, stratified for treatment (n placebo (PBO)=14; n rituximab (RTX)=12) at baseline and arthritis visit are shown for patients who developed rheumatoid arthritis (RA) within the study period. Median (IQR) time of RA onset was 12.0 (2.0–15.0) months in the PBO group and 16.5 (9.75–28.0) in the RTX group (PBO=red line; RTX=blue line).

We also did not find significant differences in HAQ-DI (mean±SEM: PBO=0.86 ± 0.20; RTX=0.93 ± 0.15), EQ-5D (mean±SEM: PBO=0.64 ± 0.05; RTX=0.63 ± 0.06), VAS pain (mean±SEM: PBO=50.29 ± 5.99; RTX=55.83 ± 6.76), PCS (mean±SEM: PBO=39.63 ± 3.08; RTX=41.14 ± 2.54) or MCS (mean±SEM: PBO=50.92 ± 1.83; RTX=48.31 ± 3.93) of the SF-36 questionnaire at the onset of arthritis between the two groups (figure 2).

## Discussion

Here, we report for the first time the PRO results of RA-risk individuals who participated in the PRAIRI study.^11^ We did not observe significant differences in the PROs of seropositive arthralgia individuals treated with RTX compared with PBO during a 2 year follow-up period. Although RA-risk individuals who received RTX had slightly worse physical functioning, reported health-related quality of life and increased pain at the start of the study, their scores were largely comparable with the general ‘healthy’ population and remained stable during the 2 year follow-up for both groups. In addition, upon arthritis development, no significant differences between RTX and PBO were observed.

The limited impact of RTX on quality of life PROs in the PRAIRI study may have several explanations. First, we cannot exclude the possibility that PRO assessments in the PRAIRI study were affected by a degree of measurement error.^19^ Patient perception of disease burden might not completely reflect disease severity and PROs validated in patients with established disease might not detect small changes in an at risk population. Minimum clinically important differences of ≥0.22 for HAQ-DI, >5.42 for PCS and>6.33 for MCS of SF-36 that were established in established RA might not be achievable in at-risk individuals, who start with less severe baseline scores than RA patients.^20^ Moreover, the sample size of the study, which was smaller than originally planned due to slow recruitment rate, might have affected the power of the analysis, and in fact a much larger population might be needed to detect significant changes in perceived quality of life. Second, a single dose of RTX might not be enough to have a significant impact on the measured PROs, whereas in a previous clinical trial of RTX in early established RA, repeated courses of RTX lead to significant improvement of PROs.^10^ Alternatively, selective targeting of B cells in RA-risk individuals might not be enough to lower the disease burden to a point that is reflected in the individual’s perception of quality of life and/or pathways involved in pain and fatigue may also be unrelated to B cells in this phase of the disease.

Individuals with clinically suspected arthralgia experience a serious disease burden even before RA onset, and an improvement in quality of life is most important for patients.^21^ PROs should be considered when evaluating preventive strategies in RA-risk individuals, and improvement in those variables should be considered as relevant as arthritis prevention. In the previously published TREAT-EARLIER trial, 1 year treatment with the conventional synthetic DMARD MTX did not prevent RA, but a significant improvement in physical functioning was observed which could be due to the pleiotropic effects of MTX.^13^ The recently published APIPPRA and ARIAA trials reported decreased development of arthritis in RA-risk individuals after the abatacept treatment (1 year and 6 months, respectively), with sustained, although less marked, differences 12 months after treatment was stopped.^12 14^ Both studies also reported improved PROs at the end of the treatment, although only the ARIAA trial reported sustained reduced pain and improved physical function and quality of life after treatment cessation. Conceivably, the difference in sustained PRO changes between the PRAIRI, TREAT-ERLIER, APIPPRA, and ARIAA studies might be due to differences in study population, as well as in the medication mechanisms of action. While the PRAIRI, APIPPRA, and ARIAA studies all included subjects seropositive for ACPA and/or RF, the TREAT-EARLIER trial included subjects regardless of their antibody status. Moreover, the APIPPRA study did not require subclinical inflammation as an inclusion criteria for study participation, while in the PRAIRI trial subjects were included only if their CRP levels were higher than 0.6 mg/L, and both the ARIAA and TREAT-EARLIER studies required subjects to have subclinical inflammation, as detected by magnetic resonance imaging. Of note, abatacept may also exert favourable effects on pain and fatigue via other mechanisms, such as tryptophan catabolism via induction of indoleamine 2,3-dioxygenase (IDO),^22 23^ which may also explain why the positive effects of abatacept on PROs are most prominent during treatment and subside after treatment cessation.

Collectively, the data from clinical trials exploring the effects of anti-rheumatic treatment administered during the ‘preventative window of opportunity’ in the preclinical phase of RA support the notion that it is possible to delay arthritis onset, but repeated treatment courses or prolonged treatment may be needed to improve PROs and/or prevent RA. As a next step, as we postulated before, one could envisage repeated treatment, perhaps with a single infusion of RTX once a year, to control B-cell numbers and prevent clinically manifest disease in RA-risk individuals or the development of therapies that target specifically autoreactive immune cells, for example, by using autoantigen-based chimeric immunoreceptors to achieve long-term prevention and improve disease symptoms from the patient’s perspective.

## supplementary material

10.1136/rmdopen-2024-004622 online supplemental table 1

## Data Availability

No data are available.
