# Supplementary material for: The effect of rituximab on patient reported outcomes in the preclinical phase of rheumatoid arthritis: 2 year data from the PRAIRI study
Source: RMD Open. 2024 Oct 18;10(4):e004622. doi: 10.1136/rmdopen-2024-004622 (PMC11492957; doi:10.1136/rmdopen-2024-004622)
Supplement: online supplemental table 1 [file rmdopen-10-4-s001.pdf]

PRO variable	Mean RTX	Mean PBO	Mean difference	95% Confidence interval
**HAQ-DI**				
Baseline	0.57	0.23	0.34	0.06 to 0.61
4 months	0.56	0.32	0.24	-0.06 to 0.54
12 months	0.63	0.35	0.24	-0.10 to 0.64
24 months	0.49	0.42	0.07	-0.33 to 0.48
**EQ-5D**				
Baseline	0.70	0.79	-0.09	-0.20 to 0.02
1 month	0.73	0.77	-0.04	-0.18 to 0.09
4 months	0.73	0.77	-0.04	-0.15 to 0.07
12 months	0.72	0.74	-0.02	-0.15 to 0.11
24 months	0.76	0.78	-0.02	-0.16 to 0.12
**VAS pain**				
Baseline	30.88	23.11	7.77	-5.79 to 21.32
1 month	32.59	27.82	4.76	-12.48 to 22.00
4 months	30.98	33.29	-2.31	-20.11 to 15.49
6 months	30.98	32.20	-1.23	-19.05 to 16.60
12 months	38.96	25.69	13.27	-4.39 to 30.93
24 months	34.29	23.17	11.11	-9.08 to 31.31
**PCS SF-36**				
Baseline	44.70	45.61	-0.91	-6.62 to 4.79
4 months	44.86	44.05	0.81	-5.49 to 7.12
12 months	45.29	44.45	0.84	-5.84 to 7.53
24 months	44.85	47.82	-2.97	-11.34 to 5.40
**MCS SF-36**				
Baseline	50.62	50.31	0.30	-5.42 to 6.03
4 months	50.02	51.43	-1.41	-6.84 to 4.02
12 months	49.63	48.89	0.74	-5.77 to 7.24
24 months	49.79	46.80	2.99	-4.54 to 10.53

Supplementary Table 1 – Table reports mean difference and 95% confidence intervals of effect of rituximab (RTX) vs placebo (PBO) on patients reported outcomes at each timepoint.
